# Routine Immediate Eye Examination at the Point of Care for Diagnosis of AIDS-Related Cytomegalovirus Retinitis Among Patients With a CD4 Count <100 in Myanmar

**DOI:** 10.1093/ofid/ofz280

**Published:** 2019-06-14

**Authors:** Win Le Shwe Sin Ei, Kyi Pyar Soe, Adelene Hilbig, Jillian Murray, David Heiden

**Affiliations:** 1 MSFCH, Yangon, Myanmar Mission, Myanmar; 2 MSFCH, Dawei, Myanmar Mission, Myanmar; 3 Medecins Sans Frontieres, Geneva, Switzerland; 4 Pacific Eye Associates, California Pacific Medical Center Department of Ophthalmology, San Francisco, California; Seva Foundation, Berkeley, California

**Keywords:** AIDS-related opportunistic infections, CMV retinitis, early diagnosis of CMV retinitis, indirect ophthalmoscopy by nonophthalmologists

## Abstract

A retrospective review of diagnosis of cytomegalovirus retinitis (CMVR) before and after introduction of routine immediate eye examination among AIDS patients in Myanmar with an absolute CD4 T-cell count <100 cells/μL demonstrated an increased detection of CMVR from 1.1% (14/1233) to 10.7% (65/608), an improvement of ~10-fold. Diagnosis of CMVR was achieved a mean of 2 days after clinic enrollment.

Cytomegalovirus retinitis (CMVR) is a common late-stage AIDS-related opportunistic infection that occurred in up to one-third of patients before the combined antiretroviral therapy (cART) era. CMVR has virtually disappeared in high-income countries but remains prevalent in middle- and low-income countries [1], as ~20% of patients first seek care with advanced HIV infection [2].

CMVR accounts for >90% of AIDS-related blindness, and due to inadequate management, a growing cadre of HIV patients are being made well by cART but left irreversibly blind from CMVR. This is highlighted by a report from Thailand, where CMVR followed only curable cataract as a cause of blindness [3], despite only occurring in HIV-positive individuals, who comprise but 1.4% of that general population.

CMVR is but 1 end-organ manifestation of CMV disease, a potentially fatal opportunistic infection [1, 4], with disseminated CMV infection being found at autopsy in up to half of patients who die of HIV/AIDS [5]. In resource-limited settings, a diagnosis of CMVR may be established on physical examination alone, whereas the diagnosis of other manifestations of CMV disease is difficult [1, 4]. Patients with CMVR have an all-cause mortality >25%, with most fatal outcomes in the first 3 months [6]. Because early diagnosis of CMVR and provision of systemic anti-CMV therapy may provide an opportunity to reduce AIDS mortality and blindness, a premium must be placed on establishing the diagnosis at the earliest possible moment.

One strategy to achieve early diagnosis of CMVR is to shift management of this opportunistic infection to the HIV/AIDS clinician, who routinely manages all other opportunistic infections. The feasibility of providing diagnosis at the point of care by nonophthalmologists has previously been demonstrated [7]. The mechanics of introducing this innovation have also been described [8], but not the impact on clinical care.

At the Medicins Sans Frontiers (MSF) HIV clinic in Dawei, Myanmar, we trained nonophthalmologist HIV clinicians to perform retinal examination by indirect ophthalmoscopy for diagnosis of CMVR at the point of care. To evaluate the impact on clinical care, we compared diagnosis of CMVR before and after introduction of routine indirect ophthalmoscopy.

## METHODS

We conducted a retrospective review of all patients diagnosed with CMVR from HIV project commencement in 2004 until the end of 2017. We identified patients with a comorbid diagnosis of CMVR from 2004 until April 2017 using MSF FUCHIA software (Epicentre, Paris, France) and conducted chart reviews to extend inclusion of CMVR patients through December 2017. The total number of patients who first presented for care with a CD4 count <100 cells/μL was recorded by date. The number of days from first presentation to the Dawei clinic until eye examination was noted.

From 2004 to January 2013, patients were presumptively diagnosed with CMVR based on low CD4 count and vision loss. Retinal examination was not routinely performed, and treatment was not available.

From February 2013, all patients with an initial CD4 count <100 cells/µL had retinal examination by indirect ophthalmoscopy through a fully dilated pupil by a nonophthalmologist HIV doctor who had been trained in this technique. The accuracy of diagnosis was monitored by an ophthalmologist who made 6 evaluation visits.

With a Fisher exact test, we compared detection of CMVR before and after implementing routine screening.

## RESULTS

From 2004 to the end of January 2013, 14/1233 (1.1%) patients who enrolled with a baseline CD4 count <100 cells/μL were diagnosed with CMVR (Table 1).

**Table 1.** Detection of CMVR Before and After Institution of Routine Eye Examination by Indirect Ophthalmoscopy

	Before Routine Eye Examination	After Routine Eye Examination
Dates	2004–January 31, 2013	February 1, 2013–December 31, 2017
No. of HIV patients with a CD4 <100	1233	608
No. of CMVR detected	14	65
Detection rate, %	1.1	10.7

From February 2013 to December 2017, 65/608 (10.7%) patients who enrolled with a baseline CD4 count <100 cells/μL were diagnosed with CMVR and subsequently treated.

The median number of days between presentation to the Dawei clinic and routine retinal examination for CMVR (interquartile range) was 2 (1–4).

The rate of detection of CMVR significantly increased with routine retinal examination (*P *< .001).

## DISCUSSION

Routine immediate retinal examinations of all patients with CD4 counts <100 cells/μL in Myanmar demonstrated increased detection of CMVR by ~10-fold. Although time trends could confound the relationship between the introduction of routine examination and detection rate, this is unlikely because cases of CMVR are thought to be decreasing, and thus the effect of screening on CMVR detection may actually be an underestimate.

The crucial step for providing better outcomes for patients with CMVR is timely diagnosis, which requires retinal examination. In Thailand [9] and India [10], both regions with numerous ophthalmologists, performance of retinal examination is reported to occur months to years after commencement of cART. A controlled study from Thailand demonstrated that late diagnosis led to less favorable visual outcomes [9], and in a report from India, 50.6% of eyes with CMVR were already blind at first diagnosis [11]. In reality, in most middle- and low-income countries, particularly in resource-limited settings, there is no access to appropriate retinal examination for patients with HIV/AIDS, with a strong pattern of late or no diagnostic evaluation for CMVR, leading to systematic inadequate clinical care. We have established that retinal examination and diagnosis of CMVR can be provided a mean of 2 days after first presenting for HIV care, even in a remote setting with virtually no access to ophthalmologists. In the Dawei clinic, early diagnosis permitted immediate treatment with either intraocular ganciclovir injection, oral valganciclovir, or both, depending upon drug availability and patient status.

## CONCLUSIONS

Our data support routine immediate retinal examination at the point of care for all HIV patients with a CD4 count <100 cells/µL in resource-limited settings in Southeast Asia. This may best be achieved by a nonophthalmologist whereever immediate diagnostic capacity for CMV retinitis is not realistically available.
